# Serum Desmosine Levels Might Be Associated with the Size of Ruptured Cerebral Aneurysms in Patients with Aneurysmal Subarachnoid Hemorrhage—A Preliminary Study

**DOI:** 10.3390/jcm14062056

**Published:** 2025-03-18

**Authors:** Peter Csecsei, Agnes Bogdan, Tihamer Molnar, Laszlo Zavori, Attila Schwarcz, Gabor Lenzser

**Affiliations:** 1 Department of Neurosurgery, Medical School, University of Pecs, 7624 Pecs, Hungary; schwarcz.attila@pte.hu (A.S.); lenzser.gabor@pte.hu (G.L.); 2 Department of Medical Biology, Medical School, University of Pecs, 7624 Pecs, Hungary; agnesbogdan@gmail.com; 3 Department of Anesthesiology and Intensive Care, Medical School, University of Pecs, 7624 Pecs, Hungary; molnar.tihamer@pte.hu; 4 Emergency Department, Saudi German Hospital, Dubai 61313, United Arab Emirates; zavori.laszlo@gmail.com

**Keywords:** aneurysmal subarachnoid hemorrhage, size of cerebral aneurysm, desmosine, size ratio

## Abstract

**Background:** Aneurysmal subarachnoid hemorrhage (aSAH) is a disease associated with high mortality, caused by the rupture of a cerebral aneurysm. Decision-support scoring systems used for managing unruptured aneurysms (UIAs) include only radiological parameters related to the size and configuration of the aneurysm, without incorporating blood-based markers. Our aim is to identify a serum marker that shows a correlation with aneurysm size in patients with ruptured aneurysms. **Methods**: Arterial blood samples were collected from patients who experienced aSAH within 24 h of the ictus, and serum desmosine levels were determined using ELISA. The morphological parameters of the aneurysms were assessed during 3D DSA. A favorable outcome was defined as a 3-month mRS score of 0–3. **Results**: This study included 135 aSAH patients and 25 controls. (i) The desmosine level in serum collected within 24 h after aneurysm rupture in patients with aSAH was significantly higher compared to the serum level in the control group (aSAH: 0.737 ng/mL [IQR: 0.401–1.214], vs. control: 0.365 ng/mL [IQR: 0.251–0.531], *p* < 0.001); (ii) examining the size of ruptured aneurysms, patients with aneurysms larger than 7 mm had significantly higher serum desmosine levels than those with aneurysms smaller than 7 mm; (iii) in the group with aneurysms smaller than 7 mm, serum desmosine levels correlated with the aneurysm neck width and the size ratio. **Conclusions**: Serum desmosine shows a strong correlation with the size of ruptured aneurysms in aSAH patients.

## 1. Introduction

Aneurysmal subarachnoid hemorrhage (aSAH), although its mortality rate shows a decreasing trend [1], is still considered a serious condition. aSAH places a significant burden on society, with an estimated incidence of 6 to 8 cases per 100,000 people per year, and morbidity remains high [2]. For these reasons, treating rupture-prone aneurysms before they bleed is critically important. Current treatment options, such as microsurgical clipping and endovascular coiling, carry specific risks, which should be carefully considered in decisions regarding unruptured aneurysms.

Several risk factors have been associated with rupture, including smoking, hypertension, certain aneurysmal morphological features (e.g., aneurysm size, daughter sacs, aspect ratio, size ratio), family history, and ethnicity [3]. Based on these, decision-support scoring systems (e.g., PHASES, UIATS) have been developed [4,5] to guide clinical decisions regarding treatment. In all of these scoring systems, the size and location of the aneurysm are important parameters; however, only one of them (UIATS) considers derived parameters (aspect ratio, size ratio), which are crucial for characterizing the aneurysm. The size and morphological characteristics of the aneurysm (AR, SR) are important risk factors for rupture [6]. Moreover, in certain locations, smaller aneurysms also carry a higher risk of rupture [6]. The aneurysm neck width and the SR have been proven to be independent risk factors for rupture status [7,8]. None of the predictive scores include a blood-measurable biomarker that could provide insights into the size or risk status of an aneurysm. Desmosine and isodesmosine are two pyridinium amino acids that act as positional isomers, functioning as crosslinking molecules to bind polymeric amino acid chains into elastin’s three-dimensional network [9]. During the degradation of elastin-containing tissues, such as in pulmonary emphysema [10], COPD [11], or abdominal aortic aneurysm [12], desmosine can appear in the urine, sputum [9], and blood. A positive association has been found between plasma desmosine levels and the degree of aortic dilation in patients with Marfan syndrome [13]. Moreover, a small-scale study observed higher plasma desmosine levels in acute stroke patients compared to the control group [14]. A study by Nakagawa et al. demonstrated a significantly higher concentration of soluble human elastin fragments in the lumen of ruptured intracranial aneurysms compared to nonruptured ones [15]. In addition, elastin degradation is considered to play an important role in the development of cerebral aneurysms [16]. Based on this, it can be hypothesized that desmosine may play a role in the formation of cerebral aneurysms, during potential rupture and may also be correlated with the size of the aneurysm. The main objective of our current study is to investigate the correlation between serum desmosine levels and clinical as well as aneurysm morphological parameters in patients with aSAH.

## 2. Materials and Methods

### 2.1. Study Design and Subjects

In this single-center, prospective study, a total of 174 patients were screened between February 2021 and April 2024. Institutional review board approval was obtained previously (IV/8468-1/2021/EKU, 27.10.2021). Of these, 135 patients met the inclusion criteria, which were as follows: (i) subarachnoid hemorrhage caused by a ruptured aneurysm, (ii) age 18 years or older, (iii) signed informed consent by the patient or their legal representative. Forty-nine patients were excluded for the following reasons: missing biomarker data (*n* = 5), malignancies (*n* = 3), COPD or other lung diseases (*n* = 2), liver and/or kidney diseases (*n* = 5), confirmed abdominal aortic aneurysm or aneurysm in other parts of the body (*n* = 4), immunological disease (6), loss of follow-up (*n* = 6), non-traumatic cause of SAH/AVM (*n* = 5), and multiple cerebral aneurysm on DSA (13). As a control group, we selected 25 healthy, non-smoking, age- and sex-matched individuals who had undergone cranial CT/MR angiography for other reasons and abdominal ultrasound, and who had previously been treated for mild post-COVID-19 symptoms.

The clinical management of patients adhered to the guidelines set by the Neurocritical Care Society [17] and the American Heart Association [18]. The patients underwent endovascular aneurysm treatment within 24 h of rupture and were placed under intensive care unit observation, where all patients received oral nimodipine and intravenous hydration with 0.9% saline, with additional fluids as necessary to maintain euvolemia. After admission, we recorded significant comorbidities, smoking, and diabetes.

The diagnosis of SAH was confirmed either by the admission CT scan or by the presence of xanthochromia in the lumbar puncture if the initial CT scan was inconclusive. A venous blood sample was collected within 24 h of admission as part of the routine clinical evaluation. Neuroradiological grading was performed using the modified Fisher scale, which categorizes the amount of subarachnoid blood on the initial CT scan as none, diffuse thin, localized thin, diffuse thick, or localized thick, along with the presence of intraventricular hemorrhage (IVH), intracerebral hemorrhage, and hydrocephalus [19]. The World Federation of Neurological Societies (WFNS) score was used for clinical group classification [20]. Delayed cerebral ischemia was diagnosed based on established guidelines [21]. A favorable outcome for 3 months was considered a modified Rankin scale score of 0–3, which was assessed 90 days (±5 days) after the ictus, either in an outpatient setting or via phone, with the assistance of an independent, trained evaluator.

### 2.2. Definition of Aneurysm Morphological Parameters

For each patient, digital subtraction angiography (DSA) images and three-dimensional (3D) reconstructions during treatment were obtained using the Philips Allura Xper FD 20/15 system. During this process, we determined the number of aneurysms, their location, aneurysm size, neck diameter, parent artery diameter, aspect ratio (AR), and size ratio (SR) [22]. Aneurysm size was defined as the maximum distance of the dome from the aneurysm neck plane. Aspect ratio (AR) was calculated from the maximum perpendicular height of the aneurysm divided by the average neck diameter of the aneurysm. Size ratio (SR) was calculated from the maximum aneurysm height divided by the mean vessel diameter of all branches associated with the aneurysm. For each patient, two independent neuroradiologists performed the measurements, and the average value was used for subsequent statistical analysis. In our study, the aneurysms were divided into six location groups: internal carotid artery (ICA), middle cerebral artery (MCA), anterior communicating artery (Acom), posterior communicating artery (Pcom), anterior cerebral artery (ACA), and vertebrobasilar (vertebral artery, basilar artery, posterior inferior cerebral artery, and superior cerebellar artery). For the size-based categorization of aneurysms, we used the thresholds defined by the PHASES score [4]. Based on this, we classified aneurysms into groups of less than 7 mm, 7–9.9 mm, and 10–19.9 mm. (There were no aneurysms larger than 20 mm in the cohort.)

### 2.3. Biomarker Measurements

Arterial blood samples were taken from patients with subarachnoid hemorrhage confirmed by native CT within 24 h after the ictus. The samples were centrifuged at 4000 rpm for 10 min, after which the serum was separated and stored at −80 degrees Celsius until analysis. For the desmosine measurement, a human desmosine ELISA kit was used (Cusabio, Houston, TX, USA, CSB-E12871h). Reagents and serum samples were prepared according to the manufacturer’s instructions. Solutions were brought to room temperature 30 min before use. Biotinylated anti-desmosine antibody and HRP-avidin were diluted by the provided diluents (100-fold dilution). Standards were prepared from a stock solution by serial dilutions (10 ng/mL desmosine, 5 ng/mL, 2.5 ng/mL, 1.25 ng/mL, 0.625 ng/mL, 0.312 ng/mL, 0.156 ng/mL), sample diluent served as blank (standard zero, 0 ng/mL desmosine). Samples were thawed on ice. A total of 100 μL of standards and samples were added to each well, and the plate was incubated for 2 h at 37 °C. After the incubation, the liquid was removed from each well, and 100 μL of biotinylated anti-desmosine antibody was added. Following incubation (1 h, 37 °C) the antibody was aspirated and the wells were washed (3 × 200 μL washing buffer). HRP-avidin was added to the wells (100 μL), and the plate was incubated for 1 h at 37 °C. After aspiration of the HRP-avidin, the wells were washed (5 × 200 μL washing buffer), then 90 μL TMB Substrate was added to each well, followed by incubation (25 min, 37 °C). The reaction was stopped by adding 50 μL Stop solution to each well, then the plate was read at 450 nm. The standard curve was plotted by four parameter logistic (4-PL) curve-fit, and the results were blank corrected (vagy blank correction was applied). A preliminary experiment was performed with a few samples at various dilutions (2-fold, 5-fold, 10-fold, and 20-fold), as well as without dilution. Based on the results of that measurement, the samples were used without dilution for the experiment.

### 2.4. Statistical Analysis

All analyses were conducted with SPSS^®^ Statistics version 25 (IBM Corporation, Armonk, NY, USA) and GraphPad Prism 9 software (GraphPad Software, San Diego, CA, USA). Continuous variables are reported as mean ± SD or median with interquartile range, whereas categorical variables are reported as number and percentage. Differences between groups were assessed using unpaired t tests, analysis of variance, Kruskal–Wallis tests, or chi-squared tests, as appropriate. Correlations were assessed using Spearman’s correlation. The accuracy of desmosine in predicting the size of aneurysm was evaluated by receiver operating characteristic curves, and the data are presented as the area under the curve (AUC).

## 3. Results

### 3.1. Patient Characteristics

**Figure 1:**
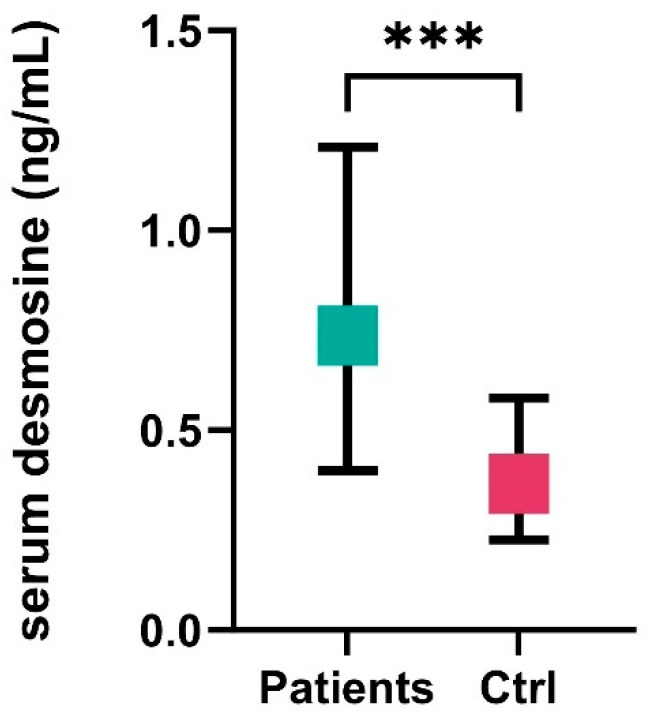
Serum desmosine levels in the patient group (*n* = 135) and the control group (*n* = 25). Ctrl, control, ***, *p* < 0.001.

A total of 135 patients were included in the analysis, with a mean age of 58.4 ± 12.4 years, and nearly three-quarters (73%) were female. The median WFNS score at admission was 2 (IQR: 1–4). The rate of favorable outcomes at 3 months (mRS 0–3) was 56%. The median size of aneurysms detected on DSA was 7 mm (IQR: 5–10.7). The most common aneurysm location was the Acom (31%), followed by the MCA at 24%. In the patient group (*n* = 135), the serum desmosine level was 0.737 ng/mL (IQR: 0.401–1.214), while in the control group (*n* = 25), it was 0.365 ng/mL (IQR: 0.251–0.531), *p* < 0.001, Figure 1. Considering the relatively smaller size of the control group, we also compared our results to a larger control group from a similar study. In the study by Mordi et al. [12], which compared patients with abdominal aortic aneurysms to those without aneurysms, the average plasma desmosine level in the control group (*n* = 162) was 0.33 ± 0.16 ng/mL. This is very similar to the levels observed in our control group [0.365 ng/mL (IQR: 0.251–0.531)] (we were unable to calculate statistical significance due to the absence of the complete dataset). No significant differences were observed in the age (years) distribution (aSAH: 58.4 ± 12.4 vs. control: 53.3 ± 8, *p* = 0.074) or gender distribution (aSAH: 73% vs. control: 66.7%, *p* = 0.366) between the patient group and the control group. The detailed parameters of the cohort are shown in Table 1.

### 3.2. Relationship Between Serum Desmosine Levels and Aneurysm Morphology

**Figure 2:**
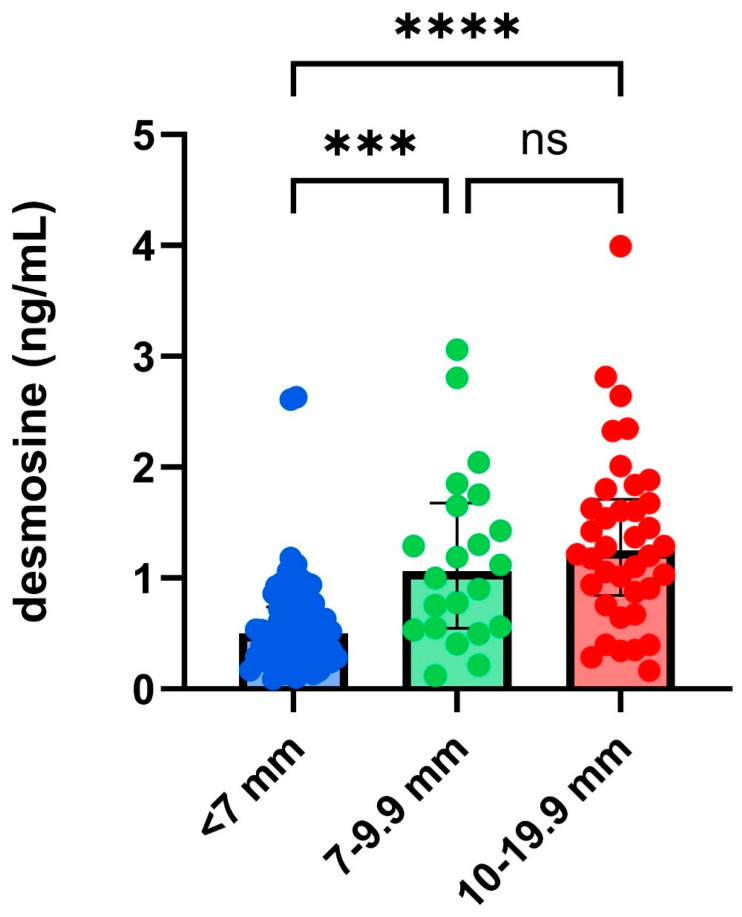
Serum desmosine levels collected 24 h after rupture, categorized by the size of the aneurysm measured on 3D-DSA. The size-based categorization of aneurysms is based on the PHASES score [4] classification. ns: non-significant; number of aneurysms in each group: <7 mm (*n* = 69), 7–9.9 mm (*n* = 25), 10–19.9 mm (*n* = 41). ***, *p* < 0.001, ****, *p* < 0.0001.

Within 24 h after rupture, the serum desmosine level was significantly higher in patients with aneurysms larger than 7 mm compared to those with aneurysms smaller than 7 mm (*p* < 0.001). No significant difference in serum desmosine levels was observed between patients with aneurysms sized 7–9.9 mm and those with aneurysms larger than 9.9 mm, as shown in Figure 2.

We examined the correlation between aneurysm morphological data and serum desmosine levels for aneurysms smaller than 7 mm and those larger than 7 mm. We found that aneurysm size showed a positive correlation with serum desmosine levels in both groups (Spearman rho, 0.327 and 0.287, respectively), while the size ratio and aneurysm neck diameter only showed a negative correlation with serum desmosine levels in aneurysms smaller than 7 mm (Spearman rho, −0.336 and −0.326, respectively). Age, admission WFNS score, CRP, and creatinine levels showed no correlation with serum desmosine levels, Table 2.

**Figure 3:**
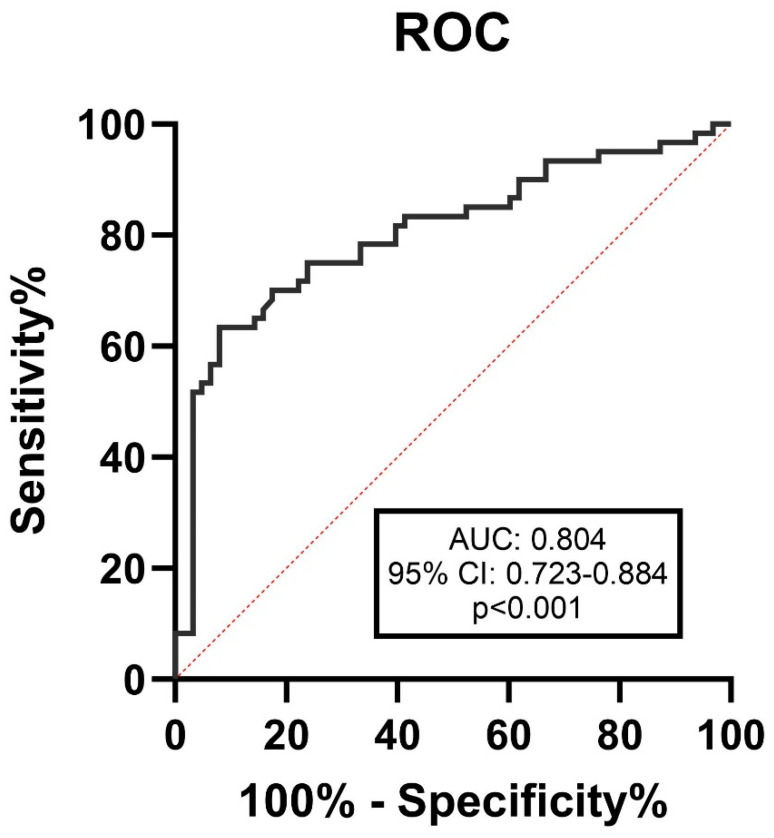
ROC curve for serum desmosine levels in detecting aneurysms larger than 7 mm. The best cut-off value was 0.533 ng/mL with the sensitivity and specificity of 81.7% and 60.3%, respectively. Red dashed line: line of equality or random chance.

The area under the curve of serum desmosine to differentiate between the large (>7 mm) and small (<7 mm) ruptured aneurysms on DSA was 0.804 (95% CI 0.723 to 0.884), *p* < 0.001, Figure 3. The cut-off value was determined to be 0.533 ng/mL, with a sensitivity of 81.7% and a specificity of 60.3%.

## 4. Discussion

In this study, we found the following results: (i) The desmosine level in serum collected within 24 h after aneurysm rupture in patients with aSAH was significantly higher compared to the serum level in the control group; (ii) examining the size of ruptured aneurysms, patients with aneurysms larger than 7 mm had significantly higher serum desmosine levels than those with aneurysms smaller than 7 mm; (iii) in the group with aneurysms smaller than 7 mm, serum desmosine levels correlated with the aneurysm neck width and the size ratio. We hypothesize that the higher serum desmosine levels observed in patients who experienced subarachnoid hemorrhage, compared to the control group, can be attributed to the breakdown of elastin released from the damaged aneurysm tissue during the rupture. Since patients with comorbidities known to increase desmosine levels (e.g., COPD, abdominal aortic aneurysm [10,11,12], etc.) were excluded from this study, the difference observed between the two groups is presumed to be associated with the rupture of the aneurysm. The outcomes differed significantly between the two groups (<7 mm vs. >7 mm), suggesting the possibility that the difference in desmosine levels between the groups might be due to the outcomes. However, serum desmosine levels showed no correlation with clinical (WFNS) or radiological (mFisher score) scales, nor with 3-month outcomes, and no association was observed with outcomes within the individual groups either. In our cohort, the significantly worse outcomes observed in patients with larger aneurysms (>7 mm) can be partially explained by the higher prevalence of MCA (middle cerebral artery) aneurysms in this group. This is associated with an increased need for EVD (external ventricular drainage), which is known to elevate the risk of unfavorable outcomes [23]. Aneurysm size itself is also associated with outcomes in cases of aSAH [24]. Plasma desmosine concentrations correlated with the maximal abdominal aortic aneurysm (AAA) diameter and log plasma desmosine concentration was associated with increased likelihood of an AAA event (death, rupture, or urgent repair) in a recent, large study with AAA patients [12]. According to this study, measuring plasma desmosine levels could be a useful additional biomarker in the therapeutic decision-making process [12]. Although this study [12] determined levels from plasma, the levels measured in serum in our study are comparable to the concentrations observed in both the control and patient groups. Considering the abdominal aortic aneurysm, it typically comprises a larger tissue mass than the aneurysms observed in our study. However, the similarity in concentrations can be attributed to the fact that we investigated patients with ruptured aneurysms. It is assumed that rupture involves more significant elastin degradation and consequently a greater increase in desmosine levels compared to stable aneurysms. Nakagawa et al. found that the mean of plasma concentration of serum soluble human elastin fragments was significantly higher in ruptured aneurysms than unruptured aneurysms [15], which also supports our findings that significantly greater elastin degradation can be measured during aneurysm rupture compared to stable aneurysms. The results of other studies also support our findings, suggesting that desmosine appears to be more specific to vascular elastin breakdown rather than lung elastin degradation [12,25]. In our current study, we excluded individuals with COPD or other chronic lung diseases, but we did not exclude smokers, given that smoking is a known risk factor for aSAH. However, we found no correlation between smoking and serum desmosine levels or aneurysm size, which aligns with previous large-scale studies conducted on patients with abdominal aortic aneurysms [12]. Although in this study we measured serum desmosine levels at only one time point, which is a limitation, a [12] previous study involving measurements at multiple time points indicates that its levels are relatively stable. This reduces the significance of this limitation in our study. Another study plasma desmosine concentrations predict clinical outcomes in patients with acute myocardial infarction (AMI), demonstrating its potential role as a prognostic marker in AMI [26]. Results from other studies examining role in desmosine in various diseases [27,28,29] suggest that desmosine predominantly reflects elastin degradation in the vascular tissue of the vascular system, likely to be caused by vascular tissue inflammation or atherosclerosis. In our study, we observed an inverse correlation between serum desmosine levels and both the neck width of the ruptured aneurysm and the size ratio in patients with aneurysms smaller than 7 mm. Kashiwazaki et al. [30], in a large-scale study, found that the size ratio, and not the absolute size, may highly predict the risk of rupture in small unruptured intracranial aneurysms. Our finding that desmosine levels correlate with the size ratio in aneurysms smaller than 7 mm strongly suggests the possibility that, if confirmed in studies with larger sample sizes, this could lead to the identification of a serum biomarker capable of influencing treatment strategies. It is important to acknowledge that the shape and size of aneurysms can change following rupture [31]; therefore, our findings are not fully applicable to unruptured aneurysms (UIAs). Another reason why our results need to be reproduced in patients with UIAs is that in unruptured aneurysms, elastin degradation is likely a much slower process, whereas in ruptured cases, the acute vascular wall disruption may lead to a significantly greater release of elastin degradation products. Thus, desmosine appears in measurable amounts in the serum of patients with ruptured aneurysms, whereas in the case of stable UIAs, lower concentrations are expected due to the prolonged release of desmosine, although there is currently no evidence to support this. van Daal et al. demonstrated that desmosine and isodesmosine (DES), two biomarkers for elastin degradation, increase in COVID-19 patients and DES levels correlated with the amount of IL-6, suggesting a key link between inflammation and pulmonary/vascular tissue damage in COVID-19 [32]. This finding underlines the importance of inflammation in vascular conditions and supports the potential role of desmosine as a biomarker for vascular integrity and damage.

Our study has several limitations. Blood samples were collected only during the acute period, so we do not know the kinetics of desmosine levels in later stages. Additionally, the control group consisted of individuals without aneurysms or other comorbidities that could affect desmosine levels, preventing us from making comparisons between cohorts of patients with ruptured (aSAH) and unruptured (UIA) aneurysms. The number of participants in the control group is low, although statistically sufficient; however, choosing a larger control group would yield statistically stronger results. Although our study identifies a significant correlation between serum desmosine levels and aneurysm size, the lack of a comparative design, such as that employed by Signorelli et al. [33], limits the ability to draw causal conclusions.

In conclusion, our findings demonstrate a strong correlation between serum desmosine levels and the size of ruptured cerebral aneurysms in patients with aSAH. This preliminary study highlights the potential of desmosine as a biomarker for aneurysm size, though further research with a comparative design and larger cohorts is required to confirm these results and explore their clinical implications.

## Figures and Tables

**Table 1:** Patient characteristics.

Variable	Total	<7 mm	>7 mm	*p*-Value
Number of patients, *n*	135	69	66	N/A
Age, years, mean ± SD	58.4 ± 12.4	57 ± 13	59 ± 12	0.360
Female, *n* (%)	99 (73)	52 (75)	47 (73)	0.518
Hypertension, *n* (%)	71 (53)	37 (54)	34 (53)	0.544
Smoking, *n* (%)	46 (34)	23 (37)	23 (38)	0.491
Diabetes, *n* (%)	14 (9)	7 (9.5)	7 (8.5)	0.584
WFNS, median (IQR)	2 (1–4)	2 (1–4)	2.5 (1–5)	0.364
mFisher score, median (IQR)	3 (2–4)	3 (2–3)	3 (2–4)	0.344
3-month mRS score, median (IQR)	3 (2–5)	2 (1–4)	4 (2–5)	0.003
3-month mRS (0–3), *n* (%)	75 (56)	48 (70)	27 (41)	0.004
Delayed cerebral ischemia, *n* (%)	25 (19)	13 (19)	12 (18)	0.482
Extraventricular drainage, *n* (%)	60 (44)	26 (37)	34 (52)	0.092
Mechanical ventilation, *n* (%)	64 (47)	24 (35)	40 (61)	0.008
creatinine ^a^, µmol/L, median (IQR)	60 (48–71)	59 (47–77)	61 (49–70)	0.677
CRP ^a^, mg/L, median (IQR)	17 (5–59)	16 (5–45)	14 (3–77)	0.745
WBC ^a^, G/L, median (IQR)	10 (8–13)	10.6 (8–13)	10 (9–12)	
Size of aneurysm, mm, median (IQR)	7 (5–10.7)	5 (4–6)	10.8 (9–13)	<0.001
Neck width, mm, median (IQR)	3.3 (2.4–4)	2.6 (2.2–3.3)	3.7 (3.3–4.8)	<0.001
Aspect ratio, median (IQR)	1.7 (1.4–2.2)	1.52 (1.2–2.1)	1.8 (1.5–2.3)	0.022
Size ratio, median (IQR)	3.8 (2.6–5)	2.72 (1.9–3.9)	4.42 (3.7–5.5)	<0.001
Daughter sac, *n* (%)	54 (44)	22 (32)	32 (55)	0.018
Location of ruptured aneurysm, *n* (%)				0.003
ICA	6 (4.8)	2 (3.3)	4 (6.8)	
MCA	33 (24)	9 (13.1)	25 (37.3)	
Acom	42 (31.2)	31 (44.3)	9 (13.6)	
Pcom	20 (14.4)	10 (14.8)	10 (15.3)	
ACA	3 (2.4)	2 (3.3)	1 (1.7)	
Vertebrobasilar	31 (23.2)	15 (21.3)	17 (25.4)	
serum desmosine, ng/mL, median (IQR)	0.64 (0.37–1.12)	0.503 (0.285–0.740)	1.196 (0.714–1.665)	<0.001
serum desmosine, ng/mL, mean ± SD	0.932	0.588 ± 0.46	1.281 ± 0.78	<0.001

^a^ on admission; WFNS: World Federation of Neurological Societies score; IQR: interquartile range; SD: standard deviation; mRS: modified Rankin scale; CRP: C-reactive protein; WBC: white blood count; ICA: internal carotid artery; MCA: middle cerebral artery; Acom: anterior communicating artery; Pcom: posterior communicating artery; ACA: anterior cerebral artery.

**Table 2:** The correlation of serum desmosine with clinical variables and aneurysm morphological parameters.

	<7 mm (*n* = 69)		>7 mm (*n* = 66)	
Variable	*ρ*	*p*-Value	*ρ*	*p*-Value
Size of aneurysm	0.327	0.009	0.287	0.026
Size ratio	−0.336	0.008	−0.006	0.966
Aspect ratio	0.145	0.266	0.046	0.731
Neck width	−0.326	0.01	−0.202	0.132
CRP	−0.003	0.450	−0.111	0.450
Creatinine	0.024	0.856	−0.029	0.838
Age	0.229	0.071	−0.116	0.380
WFNS score	0.037	0.775	−0.047	0.721
mRS score (3 month)	−0.078	0.544	0.009	0.948

*ρ*, Spearman’s rho, CRP, C-reactive protein, WFNS score, World Federation of Neurological Societies score.

## Data Availability

Data supporting the findings of this study are available upon reasonable request.

## Abbreviations

The following abbreviations are used in this manuscript:

aSAH	Aneurysmal Subarachnoid Hemorrhage
AAA	Abdominal Aortic Aneurysm
ACA	Anterior Cerebral Artery
Acom	Anterior Communicating Artery
AMI	Acute Myocardial Infarction
AR	Aspect Ratio
AUC	Area Under the Curve
COPD	Chronic Obstructive Pulmonary Disease
CRP	C-Reactive Protein
CT	Computed Tomography
DSA	Digital Subtraction Angiography
ELISA	Enzyme-Linked Immunosorbent Assay
EVD	Extraventricular Drainage
HRP	Horseradish Peroxidase
ICA	Internal Carotid Artery
IQR	Interquartile Range
IVH	Intraventricular Hemorrhage
MCA	Middle Cerebral Artery
mFisher	Modified Fisher Scale
MRI	Magnetic Resonance Imaging
mRS	Modified Rankin Scale
Pcom	Posterior Communicating Artery
PHASES	Population, Hypertension, Age, Size, Earlier Subarachnoid Hemorrhage, Site
ROC	Receiver Operating Characteristic
SAH	Subarachnoid Hemorrhage
SR	Size Ratio
SD	Standard Deviation
SPSS	Statistical Package for the Social Sciences
TMB	Tetramethylbenzidine
UIA	Unruptured Intracranial Aneurysm
UIATS	Unruptured Intracranial Aneurysm Treatment Score
WFNS	World Federation of Neurological Societies
WBC	White Blood Count

## References

[1] Bugazia S., Boshnaf M., Sreenivasan A. (2024). Subarachnoid hemorrhage mortality trends in U.S. patients with circulatory disease (1999–2020). Crit. Care Med..

[2] Vlak M.H., Algra A., Brandenburg R., Rinkel G.J. (2011). Prevalence of unruptured intracranial aneurysms, with emphasis on sex, age, comorbidity, country, and time period: A systematic review and meta-analysis. Lancet Neurol..

[3] Korja M., Lehto H., Juvela S. (2014). Lifelong rupture risk of intracranial aneurysms depends on risk factors: A prospective Finnish cohort study. Stroke.

[4] Bijlenga P., Gondar R., Schilling S., Morel S., Hirsch S., Cuony J., Corniola M.-V., Perren F., Rüfenacht D., Schaller K. (2017). PHASES Score for the Management of Intracranial Aneurysm: A Cross-Sectional Population-Based Retrospective Study. Stroke.

[5] Molenberg R., Aalbers M.W., Mazuri A., Luijckx G.J., Metzemaekers J.D.M., Groen R.J.M., Uyttenboogaart M., van Dijk J.M.C. (2021). The Unruptured Intracranial Aneurysm Treatment Score as a predictor of aneurysm growth or rupture. Eur. J. Neurol..

[6] Sanchez S., Hickerson M., Patel R.R. (2023). Morphological characteristics of ruptured brain aneurysms: A systematic literature review and meta-analysis. Stroke Vasc. Interv. Neurol..

[7] Tang X., Zhou L., Wen L., Wu Q., Leng X., Xiang J., Zhang X. (2022). Morphological and hemodynamic characteristics associated with the rupture of multiple intracranial aneurysms. Front. Neurol..

[8] Duan Z., Li Y., Guan S., Ma C., Han Y., Ren X., Wei L., Li W., Lou J., Yang Z. (2018). Morphological parameters and anatomical locations associated with rupture status of small intracranial aneurysms. Sci. Rep..

[9] Ma S., Lieberman S., Turino G.M., Lin Y.Y. (2003). The detection and quantitation of free desmosine and isodesmosine in human urine and their peptide-bound forms in sputum. Proc. Natl. Acad. Sci. USA.

[10] Cantor J. (2024). The role of the extracellular matrix in the pathogenesis and treatment of pulmonary emphysema. Int. J. Mol. Sci..

[11] Luisetti M., Ma S., Iadarola P., Stone P.J., Viglio S., Casado B., Lin Y.Y., Snider G.L., Turino G.M. (2008). Desmosine as a biomarker of elastin degradation in COPD: Current status and future directions. Eur. Respir. J..

[12] Mordi I.R., Forsythe R.O., Gellatly C., Iskandar Z., McBride O.M., Saratzis A., Chalmers R., Chin C., Bown M.J., Newby D.E. (2019). Plasma desmosine and abdominal aortic aneurysm disease. J. Am. Heart Assoc..

[13] Iskandar Z., Dodd M., Huang J., Chin C.W.L., Stuart G., Caputo M., Clayton T., Child A., Jin X.Y., Aragon-Martin J.A. (2023). Exaggerated elastin turnover in young individuals with Marfan syndrome: New insights from the AIMS trial. Eur. Heart J. Open.

[14] Mikagi A., Tashiro R., Inoue T., Anzawa R., Imura A., Tanigawa T., Ishida T., Inoue T., Niizuma K., Tominaga T. (2022). Isotope-dilution LC-MS/MS analysis of the elastin crosslinkers desmosine and isodesmosine in acute cerebral stroke patients. RSC Adv..

[15] Nakagawa D., Zanaty M., Hudson J., Teferi N., Ishii D., Allan L., Jabbour P., Ortega-Gutierrez S., Samaniego E.A., Hasan D.M. (2018). Plasma soluble human elastin fragments as an intra-aneurysmal localized biomarker for ruptured intracranial aneurysm. J. Am. Heart Assoc..

[16] Jung K.H. (2018). New pathophysiological considerations on cerebral aneurysms. Neurointervention.

[17] Diringer M.N., Bleck T.P., Hemphill J.C., Menon D., Shutter L., Vespa P., Bruder N., Connolly E.S., Citerio G., Gress D. (2011). Critical care management of patients following aneurysmal subarachnoid hemorrhage: Recommendations from the neurocritical care society’s multidisciplinary consensus conference. Neurocrit. Care.

[18] Connolly E.S., Rabinstein A.A., Carhuapoma J.R., Derdeyn C.P., Dion J., Higashida R.T., Hoh B.L., Kirkness C.J., Naidech A.M., Ogilvy C.S. (2012). Guidelines for the management of aneurysmal subarachnoid hemorrhage: A guideline for healthcare professionals from the American Heart Association/American Stroke Association. Stroke.

[19] Claassen J., Bernardini G.L., Kreiter K., Bates J., Du Y.E., Copeland D., Connolly E.S., Mayer S.A. (2001). Effect of cisternal and ventricular blood on risk of delayed cerebral ischemia after subarachnoid hemorrhage: The Fisher scale revisited. Stroke.

[20] Drake C.G. (1988). Report of World Federation of Neurological Surgeons Committee on a universal subarachnoid hemorrhage scale. J. Neurosurg..

[21] Vergouwen M.D., Vermeulen M., van Gijn J., Rinkel G.J., Wijdicks E.F., Muizelaar J.P., Mendelow A.D., Juvela S., Yonas H., Terbrugge K.G. (2010). Definition of delayed cerebral ischemia after aneurysmal subarachnoid hemorrhage as an outcome event in clinical trials and observational studies: Proposal of a multidisciplinary research group. Stroke.

[22] Mocco J., Brown R.D., Torner J.C., Capuano A.W., Fargen K.M., Raghavan M.L., Piepgras D.G., Meissner I., John H., on behalf of the International Study of Unruptured Intracranial Aneurysms Investigators (2018). Aneurysm morphology and prediction of rupture: An international study of unruptured intracranial aneurysms analysis. Neurosurgery.

[23] Göttsche J., Piffko A., Pantel T.F., Westphal M., Dührsen L., Czorlich P., Sauvigny T. (2022). Aneurysm location affects clinical course and mortality in patients with subarachnoid hemorrhage. Front. Neurol..

[24] Shiue I., Arima H., Hankey G.J., Anderson C.S., for the ACROSS Group (2011). Location and size of ruptured intracranial aneurysm and serious clinical outcomes early after subarachnoid hemorrhage: A population-based study in Australasia. Cerebrovasc. Dis..

[25] Chalmers J.D., Moffitt K.L., Suarez-Cuartin G., Sibila O., Finch S., Furrie E., Dicker A., Wrobel K., Elborn J.S., Walker B. (2017). Neutrophil elastase activity is associated with exacerbations and lung function decline in bronchiectasis. Am. J. Respir. Crit. Care Med..

[26] Ali K., Israr M.Z., Ng L.L., Mordi I., Lang C.C., Kuzmanova E., Huang J.T.-J., Choy A.-M. (2022). Plasma desmosine for prediction of outcomes after acute myocardial infarction. Front. Cardiovasc. Med..

[27] Rabinovich R.A., Miller B.E., Wrobel K., Ranjit K., Williams M.C., Drost E., Edwards L.D., Lomas D.A., Rennard S.I., Agustí A. (2016). Circulating desmosine levels do not predict emphysema progression but are associated with cardiovascular risk and mortality in COPD. Eur. Respir. J..

[28] Huang J.T.-J., Kuzmanova E., Dicker A.J., Keir H.R., Finch S., Aliberti S., Fardon T.C., Chalmers J.D. (2020). Serum desmosine is associated with long-term all-cause and cardiovascular mortality in bronchiectasis. Am. J. Respir. Crit. Care Med..

[29] Iskandar Z., Mordi I., Huang J.T.J., Newby D., Chalmers J., Bown M., Lang C., Choy A.M. (2019). Plasma desmosine, an elastin degradation product, predicts outcomes in at-risk populations. J. Am. Coll. Cardiol..

[30] Kashiwazaki D., Kuroda S. (2013). Size ratio can highly predict rupture risk in intracranial small (<5 mm) aneurysms. Stroke.

[31] Chien A., Sayre J., Viñuela F. (2011). Comparative morphological analysis of the geometry of ruptured and unruptured aneurysms. Neurosurgery.

[32] Visser M.P.J., Dofferhoff A.S.M., van den Ouweland J.M.W., van Daal H., Kramers C., Schurgers L.J., Janssen R., Walk J. (2022). Effects of Vitamin D and K on Interleukin-6 in COVID-19. Front. Nutr..

[33] Signorelli F., Pop R., Ganau M., Cebula H., Scibilia A., Gallinaro P., Zaed I., Todeschi J., Lefevre E., Nannavecchia B. (2020). Endovascular versus surgical treatment for improvement of oculomotor nerve palsy caused by unruptured posterior communicating artery aneurysms. J. Neurointerv. Surg..

